# Nonlinear relationships between fatigue, fear of COVID-19, and PTSD among mental health professionals: the findings of a multi-site survey in China

**DOI:** 10.3389/fpsyt.2026.1731508

**Published:** 2026-04-10

**Authors:** Wei Zhang, He-Li Sun, Pan Chen, Qinge Zhang, Sha Sha, Zhaohui Su, Teris Cheung, Gabor S. Ungvari, Todd Jackson, Feng-Rong An, Yu-Tao Xiang, Gang Wang

**Affiliations:** 1 Unit of Psychiatry, Department of Public Health and Medicinal Administration, & Institute of Translational Medicine, Faculty of Health Sciences, University of Macau, Macao, Macao SAR, China; 2 Centre for Cognitive and Brain Sciences, University of Macau, Macao, Macao SAR, China; 3 College of Acupuncture-Moxibustion and Tuina, International Institute for Innovation in Acupuncture, Beijing University of Chinese Medicine, Beijing, China; 4 Department of Nursing, Chinese Academy of Medical Sciences - Peking Union Medical College, Peking Union Medical College Hospital, Beijing, China; 5 Beijing Key Laboratory of Mental Disorders, National Clinical Research Center for Mental Disorders & National Center for Mental Disorders, Beijing Anding Hospital, Capital Medical University, Beijing, China; 6 School of Public Health, Southeast University, Nanjing, China; 7 School of Nursing, Hong Kong Polytechnic University, Hong Kong, Hong Kong SAR, China; 8 Section of Psychiatry, University of Notre Dame Australia, Fremantle, WA, Australia; 9 Division of Psychiatry, School of Medicine, University of Western Australia, Perth, WA, Australia; 10 Department of Psychology, University of Macau, Macao, Macao SAR, China

**Keywords:** fatigue, fear of COVID-19, mental health professionals, non-linear relationships, PTSD

## Abstract

**Background:**

Mental health professionals (MHPs) are susceptible to fatigue, particularly during public health crises like the COVID-19 pandemic. This study examined nonlinear relationships between fatigue, post-traumatic stress disorder (PTSD), and fear of COVID-19 (FOC) among MHPs.

**Methods:**

A multi-site survey was conducted from January to February 2023. Fatigue was assessed using the Fatigue Visual Analogue Scale (VAS), PTSD with the Post-Traumatic Stress Disorder Checklist for Civilians (PCL-C), and FOC with the Fear of COVID-19 Scale (FCV-19S). Data were analyzed using logistic regression and restricted cubic splines to explore non-linear associations.

**Results:**

Of the 9,858 COVID-recovered MHPs, the prevalence of significant PTSD symptoms (PCL-17 ≥ 50) was 6.85% (95% CI: 6.35% - 7.35%), while significant fear of COVID-19 (FOC ≥ 16) was observed in 61.28% (95% CI: 60.32% - 62.24%). Higher fatigue levels were significantly associated with increased odds for exacerbated PTSD symptomatology (OR = 1.75, 95% CI: 1.65 - 1.86, *p* < 0.001) and FOC severity (OR = 1.19, 95% CI: 1.16 - 1.21, *p* < 0.001). Restricted cubic splines analysis revealed nonlinear relationships. Specifically, as fatigue rose towards an inflection point of 5.00, its association with PTSD symptoms strengthened, while its association with FOC showed a decelerating growth.

**Conclusion:**

This study underscored fatigue as a factor significantly associated with COVID-recovered MHPs, particularly regarding the presence of PTSD and FOC. However, due to the cross-sectional study design, the direction of causality between fatigue, PTSD, and FOC could not be determined. Regular monitoring and targeted interventions are crucial for managing fatigue during public health crises. Healthcare organizations should provide appropriate work-rest schedules and supportive policies during such periods.

## 1 Introduction

During the COVID-19 pandemic, mental health professionals (MHPs) provided phone counseling, online therapy, and psychological first aid to help the public cope with psychological stress and alleviate anxiety (1). Fatigue is the subjective lack of physical and/or mental energy that may contribute to declines in physical health, emotional distress, cognitive decline and reduced vigilance (2–4). MHPs experience heightened fatigue during public health crises as a result of prolonged physical activity and cognitive activity (e.g., concentration) (5), long-term exposure to highly stressful, emotionally-taxing environments (6), and demands for empathy and care of patients in need (7).

According to a systematic review and meta-analysis (8), the prevalence of post-pandemic post-traumatic stress disorder symptoms (PTSD hereafter) among healthcare workers was significantly higher than rates for infected patients and the general public. PTSD includes intrusive recollections of traumatic memories, persistent negative mood, hypervigilance, and avoidance of trauma-related situations (9). MHPs were exposed to high-risk environments of infection for extended periods in addition to high risks of infection, illness, death and social discrimination, each of which could contribute to PTSD (10, 11).

Fear of COVID-19 (FOC) is another major source of psychological stress MHPs wrestled with during the pandemic. Long-term exposure to infection risks significantly increased concerns about personal health and the risk of virus transmission to families, friends, and colleagues (8). Several studies have found that FOC is closely related to fatigue indexes including burnout and emotional exhaustion (12, 13). For instance, evidence proves that pandemic-related anxiety can mediate the relationship between infection fear and fatigue (14). These findings establish FOC as a clinically relevant variable in understanding pandemic-related psychological strain.

Previous research found that PTSD is closely associated with fatigue and burnout among MHPs (15). On one hand, PTSD symptoms are associated with fatigue and mediate the relationship between stress and fatigue, with even subclinical levels of PTSD affecting fatigue (16). On the other hand, individuals with chronic fatigue syndrome (CFS) are more likely to develop PTSD (17). Among MHPs who returned to work after recovering from COVID-19, fatigue, PTSD symptoms and FOC often persist and substantially impair quality of life (18, 19). A systematic review found that nearly all participants infected with COVID-19 reported COVID-19-related fatigue (20), and approximately half of survivors continue to exhibit significant FOC six months post-infection (21). Together, these studies illustrate the importance of attending to rather than ignoring MHPs who have recovered from COVID-19 and returned to work.

Although existing research has documented the broad impact of COVID-19 on the mental health of MHPs, investigations of the relationships among fatigue, PTSD and FOC have relied on a largely untested linear assumption. However, in a state of high fatigue, immune system sensitivity to stress responses decreases, which can lead to highly fatigued individuals showing different PTSD and FOC responses when faced with stress (22). For example, among Gulf War veterans, the prevalence of PTSD increased linearly with increasing stress intensity. However, illness resembling chronic fatigue syndrome (CFS), a condition characterized by persistent, extreme fatigue that may not improve with rest, increased only at the low end of the stress spectrum (23, 24). In addition, relations between fatigue and FOC may also be nonlinear. For example, nursing home staff with both low and severe emotional exhaustion had the highest FOC levels in one study (25).

Therefore, notwithstanding possible bidirectional relationships between fatigue, PTSD, and FOC (16, 17, 26), this study used fatigue as a variable of interest. By considering fatigue as a factor associated with further psychological distress, it is possible to explore its nonlinear associations with PTSD and FOC. To test and elucidate the nature of these links more fully, this study explored non-linear relationships between fatigue, PTSD and FOC among MHPs.

## 2 Method

### 2.1 Study design and participants

This study was based on a large, multi-site survey conducted from January 22, 2023 to February 10, 2023 (shortly after the cessation of China’s dynamic zero-COVID policy). Participants were recruited using a snowball sampling method with help from the Chinese Society of Psychiatry and the Chinese Nursing Association - Psychiatry Branch. To reduce potential risk of infection during the COVID-19 pandemic, the WeChat-embedded “Wenjuanxing” application (Changsha Ranxing Information Technology Co., Ltd., Hunan, China) was used in data collection, following previous studies (27, 28). All MHPs were required to report their health status through Wechat daily nationwide during the study period; therefore, all MHPs were presumably active WeChat users (29). Survey invitations and questionnaires linked to a QR code were distributed to all public psychiatric hospital nationwide. Prior to participation, participants were informed about the study’s purpose and procedures via the “Wenjuanxing” mini-program and provided electronic consent within the platform. Eligible participants included psychiatrists, nurses, and technicians who met the following inclusion criteria: 1) aged 18 years old or above; 2) employment in public psychiatric hospitals or psychiatric departments of general hospitals during the study period; 3) self-reported a history of COVID-19 infection; and 4) ability to understand Chinese and provide electronic written informed consent. Individuals with cognitive impairments that hinder their ability to comprehend the survey were excluded. This study protocol was approved by the Ethics Committee of Beijing Anding Hospital, China.

### 2.2 Measures

History of COVID-19 infection was assessed based on self-report using a single-item question: “Since the end of 2019, have you been infected with COVID-19?” with response options of 0 = no infection, 1 = infected without hospitalization, and 2 = infected with hospitalization. Participants who selected responses 1 or 2 were considered to have a history of COVID-19 infection and were eligible for inclusion provided that they reported full recovery and absence of acute symptoms at the time of the survey. This broad grouping based on infection history alone is heterogeneous, and includes individuals at varying stages post-infection including those experiencing ongoing post-acute symptoms. Socio-demographic and clinical characteristics of participants (age, gender, marital status, educational attainment, perceived health status, infection duration and working years) were assessed.

Following previous studies (30, 31), fatigue was measured using a fatigue visual analog scale (VAS) that included 0 (no fatigue) and 10 (extreme fatigue) as anchors. A Chinese version of the fatigue VAS was translated and successfully validated in previous research (31). Based on established criteria (32), fatigue levels were defined as follows: mild (score < 4), moderate (score ≥ 4), and severe (score ≥ 7) and were considered suitable for Chinese populations. The fatigue VAS score can also be analyzed as a continuous variable, as supported by previous research (33). The VAS was chosen for operational simplicity and brevity, as studies using VAS and brief fatigue measures have demonstrated reliability and validity comparable to comprehensive scales (34). The fatigue VAS typically demonstrates good to excellent test-retest reliability (35).

PTSD was assessed using a modified Chinese version of the Post-Traumatic Stress Disorder Checklist for Civilians (PCL-17) (36, 37) developed according to DSM-IV diagnostic criteria. The PCL-C covers PTSD domains of intrusive symptoms, avoidance/numbing symptoms, and hyper-arousal symptoms with 17 items, rated on a 5-point Likert scale ranging from 1 (“Not at all”) to 5 (“Extremely severe”). In this study, COVID-19 was used as the traumatic event. Total scores ranged from 17 to 85, with higher scores indicating more severe PTSD symptoms. A cut-off score of 50 or above was used to indicate significant PTSD symptoms (38), and it has been validated in Chinese populations (39). Empirical evidence has demonstrated a superior model fit of the PCL-17 relative to the standard DSM-5 specification (40).

The validated Chinese version of the fear of COVID-19 Scale (FCV-19S) (41, 42) was used to measure FOC. This scale comprises seven items, including: 1) Afraid of COVID-19, 2) Uncomfortable to think about COVID-19, 3) Clammy when thinking about COVID-19, 4) Afraid of losing life because of COVID-19, 5) Nervous when watching news about COVID-19, 6) Sleep difficulties caused by worry about COVID-19, and 7) Palpitations when thinking about COVID-19. Each item was rated on a five-point Likert scale from 1 (“strongly disagree”) to 5 (“strongly agree”), providing a total score range from 7 to 35. Higher scores indicated greater FOC; following previous studies (43, 44), a score of 16 or above was the threshold for identifying significant FOC. This threshold has been validated in Chinese populations.

### 2.3 Statistical analysis

Given the cross-sectional nature of the survey, the following analyses examined associations between variables, and could not establish causal relationships or directionality. All analyses were conducted using R version 4.4.1 (45). The Anderson-Darling test was used to assess normality due to its sensitivity in large samples (46). To compare socio-demographic and clinical characteristics of higher versus lower FOC subgroups and higher versus lower PTSD symptom subgroups, independent t-tests were used for normally distributed variables and Mann-Whitney U tests were applied for non-normally distributed continuous variables. Chi-square tests were used for categorical variables.

Logistic regression models were fitted using the “glm” function to assess associations of fatigue with presence of significant PTSD symptoms (PCL-17 ≥ 50) and FOC (FOC ≥ 16) (47). Following previous research (48), the first model did not control for covariates (i.e., significant group differences on background measures in univariate analysis), while the second model controlled for significant sociodemographic covariates. Odds ratios (OR), p-values, and 95% confidence intervals (CI) were calculated for each model. Additionally, sensitivity analyses were performed using linear regression with continuous PTSD and FOC scores to verify the robustness of the findings.

Restricted cubic splines (RCS), applied using the “rms” package (49), were used to capture potential non-linear relationships between (1) fatigue and PTSD and (2) fatigue and FOC (50). Following previous studies (51–53), the inflection point represented a significant change and was identified using segmented regression with the “segmented” package (54).

Following previous research (55), 3 to 5 knots were deemed appropriate for modeling nonlinear relationships. The 3-knot model used quantiles at Q10, Q50, and Q90, while 4-knot model used quantiles at Q05, Q35, Q65, and Q95, and the 5-knot model applied quantiles at Q05, Q27.5, Q50, Q72.5, and Q95 (56). To determine the optimal number of knots, a comparison of 3 to 5 knots was conducted using Akaike Information Criterion (AIC) to assess model performance. AIC was used to evaluate model fits, with the lowest value indicating the best balance between goodness of fit and model complexity (57). The knot specification yielding the best performance was selected for the final analysis (58). Furthermore, stratified analyses by all covariates were performed to examine the stability of the associations. Statistical significance was set at p < 0.05 (two-tailed) for all comparisons.

## 3 Results

### 3.1 Socio-demographic characteristics

Altogether, 11,760 mental health professionals were invited to participate in this survey. Of these, 789 reported no history of COVID-19 infection and were excluded, 1,113 declined to participate, and the remaining 9,858 met all inclusion criteria and were included in the analyses. Demographic and clinical characteristics of participants are shown in **Table 1**. **Supplementary Table 1** presents a comparison of the basic characteristics between the included and excluded participants.

**Table 1:** The impact of demographics and clinical characteristics on PTSD and fear of COVID-19 among mental health professionals.

Measure	Total (n = 9,858)	PTSD (PCL-17 ≥ 50)		Univariable analysis		Fear of COVID-19 (FOC ≥ 16)		Univariable analysis	
		Yes (n=285)	No (n=9,573)			Yes (n=6,041)	No (n=3,817)		
	n (%)	n (%)	n (%)	χ^2^	*p*	n (%)	n (%)	χ^2^	*p*
Male gender	1,750 (17.75)	78 (27.37)	1,672 (17.47)	18.59	<0.001	1,034 (17.12)	716 (18.76)	4.32	0.038
Married marital status	7,209 (73.13)	219 (76.84)	6,990 (73.02)	2.06	0.151	4,471 (74.01)	2,738 (71.73)	6.18	0.013
College or above education level	9,346 (94.81)	266 (93.33)	9,080 (94.85)	1.29	0.255	5,680 (94.02)	3,666 (96.04)	19.38	<0.001
Perceived Health Status				326.69	<0.001			164.70	<0.001
Poor	653 (6.62)	91 (31.93)	562 (5.87)			495 (8.19)	158 (4.14)		
Fair	7,043 (71.44)	181 (63.51)	6,862 (71.68)			4,444 (73.56)	2,599 (68.09)		
Good	2,162 (21.93)	13 (4.56)	2,149 (22.45)			1,102 (18.24)	1,060 (27.77)		
	Mean (SD)	Mean (SD)	Mean (SD)	*Z*	*p*	Mean (SD)	Mean (SD)	*Z*	*p*
Age (years)	34.82 (8.30)	35.99 (8.08)	34.79 (8.31)	-2.90	0.004	34.92 (8.30)	34.66 (8.30)	-1.67	0.094
Fatigue	3.98 (2.44)	7.38 (1.95)	3.88 (2.38)	-21.02	<0.001	4.39 (2.41)	3.32 (2.33)	-21.65	<0.001
Infection Duration (weeks)	2.42 (3.12)	3.54 (5.18)	2.39 (3.03)	-6.95	<0.001	2.56 (3.34)	2.20 (2.71)	-7.73	<0.001
Working Years (years)	12.65 (9.07)	13.65 (8.77)	12.62 (9.08)	-2.56	0.010	12.73 (9.01)	12.52 (9.18)	-1.71	0.087

COVID, Coronavirus Disease; FOC, Fear of COVID-19 Infection; PCL, Post-Traumatic Stress Disorder Checklist; PTSD, Post-Traumatic Stress Disorder; SD, Standard Deviation; Z, Standardized test statistic from the Mann-Whitney U Test; χ^2^, Chi-Square Test.

The mean age of the sample was 34.82 years (SD = 8.30) and a minority (17.75%) of respondents were males. On average, participants reported a moderate level of fatigue (see **Table 1**). For PTSD (PCL-17 ≥ 50), 675 participants (6.85%, 95% CI: 6.35% - 7.35%) met or exceeded the cut-off. For FOC ≥ 16, 6,041 participants (61.28%, 95% CI: 60.32% - 62.24%) had significant FOC. The mean fatigue score, measured on a 0–10 VAS, was 3.98 (SD = 2.44) and showed an approximately normal distribution (skewness = 0.32, kurtosis = -0.56) with minimal floor (6.86%) and ceiling (2.24%) effects.

### 3.2 Associations between fatigue, PTSD and FOC

In univariable analyses (**Table 1**), participants with significant PTSD symptoms (PCL-17 ≥ 50) had significantly higher fatigue scores (M = 7.38, SD = 1.95, on a 0–10 scale) compared to those with less PTSD symptomatology (M = 3.88, SD = 2.38; Z = -21.02, p < 0.001). Similarly, participants with elevated FOC (FOC ≥ 16) had significantly higher fatigue scores (M = 4.39, SD = 2.41) compared to those with lower FOC levels (M = 3.32, SD = 2.33; Z = -21.65, p < 0.001).

As shown in **Table 2**, logistic regression analyses based on unadjusted models (Models 1a and 1b) indicated that fatigue was significantly associated with increased odds for both significant PTSD symptoms (OR = 1.86, 95% CI: 1.76 - 1.98, p < 0.001) and elevated FOC (OR = 1.21, 95% CI: 1.19 - 1.23, p < 0.001). After adjusting for covariates, fatigue remained significantly associated with significant elevations in PTSD symptoms (Model 2a: OR = 1.75, 95% CI: 1.65 - 1.86, p < 0.001) and FOC (Model 2b: OR = 1.19, 95% CI: 1.16 - 1.21, p < 0.001) point made several times previously. Sensitivity analyses using linear regression with continuous variables yielded consistent results (**Supplementary Table 2**).

**Table 2:** Logistic regression models between fatigue, PTSD and fear of COVID-19 .

Model	PTSD (PCL-17 ≥ 50)			Model	Fear of COVID-19 (FOC ≥ 16)		
	*p*	*OR*	95% *CI*		*p*	*OR*	95% *CI*
Model1a	<0.001	1.86	1.76-1.98	Model1b	<0.001	1.21	1.19-1.23
Model2a	<0.001	1.75	1.65-1.86	Model2b	<0.001	1.19	1.16-1.21
Fatigue < 5.00	0.106	1.66	0.90-3.07	Fatigue < 5.00	<0.001	1.25	1.21-1.29
Fatigue ≥ 5.00	<0.001	1.61	1.49-1.74	Fatigue ≥ 5.00	0.042	1.08	1.00-1.16

Model 1b: Adjusted for no covariates;

Model 2b: Adjusted for gender, perceived health status, age, working years and infection duration.

Fear of COVID-19:

Model 1a: Adjusted for no covariates;

Model 2a: Adjusted for gender, marital status, education level, perceived health status and infection duration.

### 3.3 Non-linear relationship between fatigue, PTSD and FOC

For the FOC model, the 5-knot model (Q05, Q27.5, Q50, Q72.5, Q95) showed the lowest AIC value (AIC = 12621.71), indicating a good model fit. For the PTSD model, the 3-knot model (Q10, Q50, Q90) had the lowest AIC value (AIC = 1960.52).

**Figure 1:**
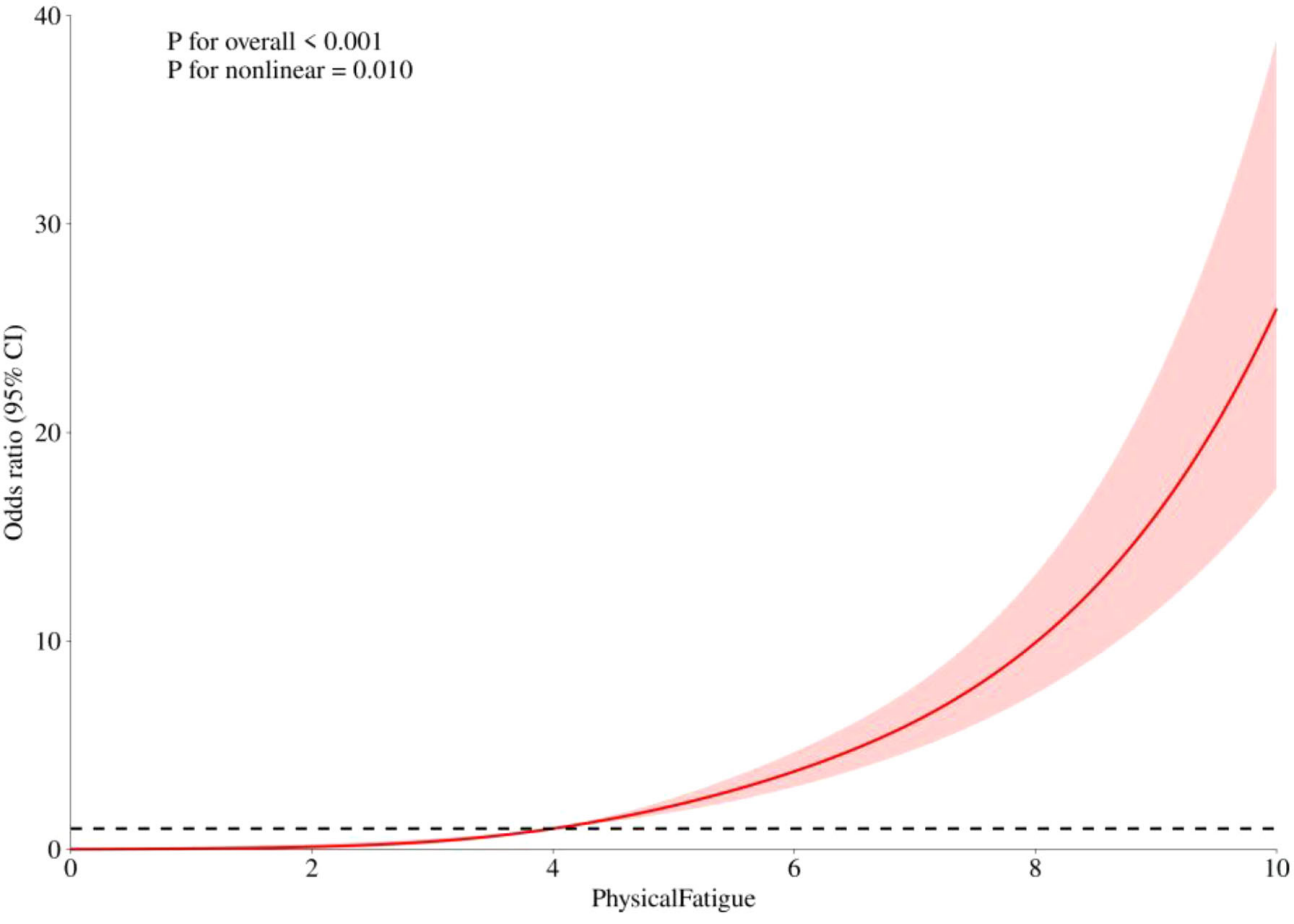
Nonlinear association of fatigue and PTSD risk. ORs with 95% CIs for PTSD risk are shown as a function of fatigue, modeled with restricted cubic splines. Both the overall association (*p* < 0.001) and the nonlinear association (*p* = 0.010) were statistically significant.

**Figure 2:**
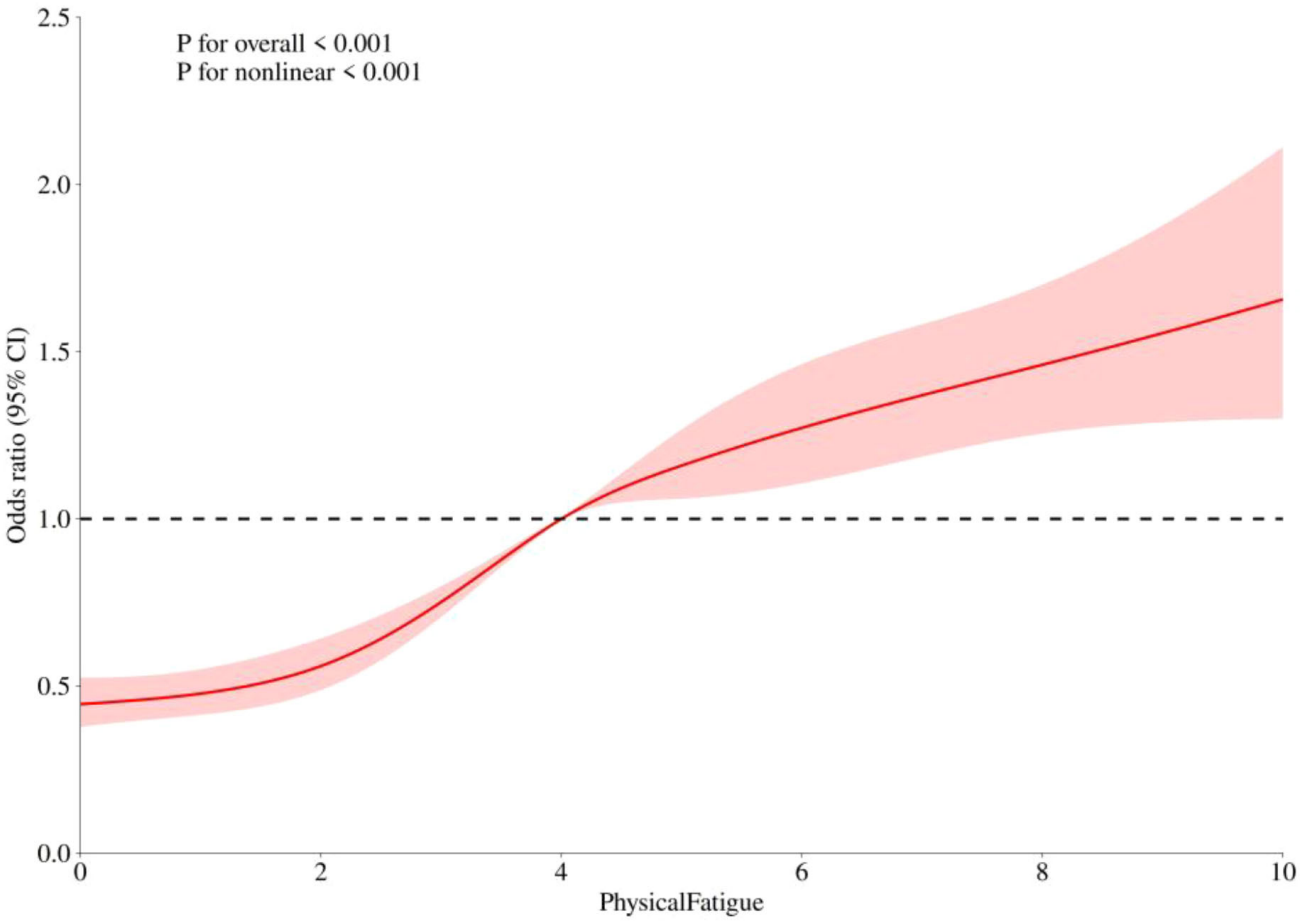
Nonlinear association of fatigue and fear of COVID-19. ORs with 95% CIs for FOC risk are shown as a function of fatigue, modeled with restricted cubic splines. Both the overall and nonlinear associations were significant (*p* < 0.001).

**Figures 1** and **2** show associations between fatigue and risk of elevated (1) PTSD symptoms and (2) FOC using restricted cubic splines with different knots, adjusted for relevant sociodemographic covariates. In both **Figures 1** and **2**, the inflection point was 5.00, which indicates a change in the slope of the risk trajectory, rather than a clinically validated cutoff. Three knots (Q10, Q50, Q90) were used for PTSD in **Figure 1**, showing a J-shaped relationship with a p value for the overall association of <0.001 and a p value for nonlinearity of 0.010. When the fatigue level was below 5.00, the OR for fatigue was 1.66 (95% CI: 0.90-3.07, p = 0.106), which did not reach statistical significance. When the fatigue level was 5.00 or higher, the OR for fatigue was 1.61 (95% CI: 1.49-1.74, p < 0.001), indicating that each unit increase in fatigue corresponded to significantly increased risk for PTSD symptom elevations by 61%. The overlapping confidence intervals suggest a gradual increase, rather than a strict cutoff. However, this sharp rise in risk makes clinical sense, as severe fatigue correlates with mental health vulnerability.

In contrast, 5 knots (Q05, Q27.5, Q50, Q72.5, Q95) were used for FOC (**Figure 2**) and showed an S-shaped relationship with both the overall association and nonlinearity; related p values were <0.001. When the fatigue level was below 5.00, the OR for fatigue was 1.25 (95% CI: 1.21-1.29, p < 0.001), indicating that for each unit increase in fatigue, the odds of FOC elevations increased by 25%. Conversely, when the fatigue level was 4.99 or higher, the OR for fatigue was 1.08 (95% CI: 1.00-1.16, p = 0.042) and the odds for FOC elevations was attenuated and increased by only 8%.

Additional stratified analyses were conducted to evaluate the robustness of these findings across different subgroups (**Supplementary Figures 1** and **S2**). For PTSD, stratified analyses by all covariates did not reveal any significant subgroups (**Supplementary Figure 1**). In contrast, for FOC, significant differences in the association between fatigue and FOC were observed based on age and working years (**Supplementary Figure 2**). Specifically, for age (P for interaction = 0.025), the OR was 1.18 (95% CI: 1.15–1.21) for participants below the median age and 1.23 (95% CI: 1.20–1.26) for those above the median age. For working years (P for interaction = 0.017), the OR was 1.18 (95% CI: 1.14–1.21) for those below the median working years and 1.23 (95% CI: 1.20–1.26) for those above the median.

## 4 Discussion

To the best of our knowledge, this is the first study to examine nonlinear relationships between fatigue, PTSD, and FOC within a healthcare sample during a public health crisis, specifically COVID-recovered MHPs. While the cross-sectional design precludes causal inferences, our analyses revealed significant and independent associations. Restricted cubic spline models demonstrated that these associations were not linear. Specifically, the relationship between fatigue (measured on a 0–10 scale) and PTSD symptom risk followed a J-shaped curve. Below the inflection point of 5.00, the association was weak; beyond this point, each unit increase in fatigue was associated with a sharply elevated risk of PTSD. In contrast, the association between fatigue and FOC severity exhibited a different pattern, resembling an S-shaped curve, whereby the relationship was strongest at lower to moderate fatigue levels and plateaued at higher levels. These findings align with and extend the results of our logistic regression models, indicating that fatigue is a significant marker for both PTSD and FOC, but its clinical significance differs qualitatively depending on severity level.

The curve for fatigue levels gradually increased up to the inflection point of 5.00 (on a 0-10 scale), beyond which its association with elevated PTSD symptom risk rose significantly. Clinically, this inflection point serves as a potential threshold for screening, which suggests that fatigue scores below 5.0 may represent manageable, non-pathological tiredness, whereas scores exceeding this level signal a pathological state where fatigue acts as a potent correlate of traumatic stress. This study suggested that fatigue is related to a reduce capacity to cope with COVID-19 as a traumatic event, as evidenced by reports of corresponding elevations in PTSD symptoms (59). These results extend previous work that reported positive linear correlations between PTSD severity and fatigue or burnout in psychiatric nurses (15). The observed nonlinear pattern may be interpreted in the context of proposed physiological mechanisms. Previous research indicates that fatigue can dysregulate the sympathetic nervous system and HPA axis, initially elevating cortisol but eventually blunting its production through heightened negative feedback (60). Our finding that PTSD risk increased markedly only after fatigue passed a threshold aligns partly with observations that lower fatigue levels may not substantially alter HPA activity (60). It is possible that sustained fatigue promotes HPA axis activation, which, in turn, could strengthen traumatic memories and exacerbate PTSD symptoms (e.g., intrusive memories) (60, 61). These findings underscore the importance of managing and reducing fatigue in PTSD interventions.

Fatigue was also positively associated with FOC in this study, consistent with previous findings (12, 13) wherein healthcare workers with higher levels of fatigue were more likely to have heightened fears of the pandemic. However, the positive fatigue-FOC relationship was more pronounced for MHPs who reported lower fatigue levels. When fatigue levels exceeded the inflection point of 5.00, their association with increases in FOC severity remained positive but was comparatively attenuated, perhaps because fatigue may be associated with reduced motivation to continue interacting with the outside world and responsiveness to external threats (62), such as COVID-19. Thus, the flattening of the curve and the small ORs at high fatigue levels should not be interpreted as a reduced risk, but as a possible indication that the nature of the fear response has changed due to cognitive or emotional exhaustion. In addition, fatigue is often accompanied by a decrease in cognitive alertness caused by long-term participation in high-cognitive demand tasks (63, 64) that may be associated with reduced ability to perceive threats (e.g., COVID-19) or reacting to threats with physiological and/or cognitive fear responses. However, this attenuation should be interpreted with caution, as alternative explanations, such as ceiling effects or unmeasured confounding factors, may also account for the findings (65). This interpretation is consistent with results of a five-year study wherein increased fatigue and persistent fatigue were associated with more rapid declines in cognitive function and daily function (66). Moreover, studies on CFS have also found CFS patients perform poorly in inferring their own emotions (67). This finding provides additional support for the hypothesis that heightened fatigue may be associated with reduced typical emotional reactions to COVID-19 among MHPs.

Together, our findings suggest that fatigue levels should be tracked among COVID-19-recovered MHPs and targeted interventions should be offered to those who are affected as a means of reducing the severity of impairing symptoms of PTSD and FOC. For instance, MHPs with comparatively mild fatigue (fatigue score < 4) may be especially prone to increased FOC, whereas those experiencing moderate to severe fatigue (fatigue score ≥ 4) are more susceptible to more severe PTSD symptoms. Therefore, it is recommended that MHPs be encouraged to regularly self-monitor changes in their fatigue levels, especially when fatigue exceeds a threshold of 4.99, and targeted interventions should be provided where necessary to address both PTSD and FOC symptoms. Educational workshops have been shown to enhance MHPs’ understanding and confidence in managing fatigue and CFS, especially emphasizing the impacts of circadian rhythm disruption (68, 69). Energy management education can also help MHPs identify and adjust activities to reduce the impact of fatigue on daily life, cultivating appropriate knowledge, skills, and attitudes, which enable them to more effectively manage their energy and activity-rest ratios (70, 71). In addition, government funding bodies and healthcare organizations should support effective fatigue management by arranging work and rest time appropriately and implementing supportive policies (e.g., sufficient staffing) to ensure that MHPs get adequate rest (72). Such strategies may help to reduce symptoms of PTSD and FOC in COVID-recovered MHPs.

A key strength of this study included its novel focus on evaluating nonlinear relationships between fatigue, PTSD and FOC among COVID-recovered MHPs through restricted cubic spline models (50). In addition, the study included a multi-site sample, increasing the likelihood that results were reliable and stable (73) as well as externally valid within a Chinese context. However, this study has several limitations. First, due to the non-experimental cross-sectional study design, causal relationships could not be established (74). Second, this study did not assess key occupational and clinical variables (e.g., professional role, work hours or shift work, region and hospital type, pre-existing psychiatric history, or prior trauma exposure), nor could we track ongoing post-acute symptoms, time since COVID-19 infection, or acute illness severity. These unmeasured factors may have confounded the observed associations between fatigue and PTSD and fear of COVID-19 among MHPs (75). Third, the use of snowball sampling and the inclusion of only non-hospitalized MHPs may have reduced representativeness of the sample and lead to an over-representation of certain subgroups (e.g., younger, more digitally-engaged individuals, or those from specific regions or hospital levels) (76, 77). Fourth, because this was an anonymous, voluntary survey, information on individuals who did not respond or declined participation was not available, which limited our ability to assess potential selection bias (78). Finally, reliance on self-report measures may have introduced reporting biases, including memory distortions, socially desirable responding, shared method variance and mood-congruent reporting, potentially influencing the observed associations (79–81).

In conclusion, this study indicated that, although fatigue has significant positive associations with severity of PTSD symptoms and FOC among COVID-recovered MHPs, such associations are not strictly linear. Evaluations of nonlinear relations indicated high levels of fatigue have a particularly strong association with PTSD symptom elevations while lower levels of fatigue are related more strongly to increases in FOC severity. Nuances in our data underscore the value of regularly monitoring fatigue and testing the efficacy of targeted interventions guided, in part, by current fatigue as a means of reducing risk for escalations in severe symptoms of PTSD and/or FOC among MHPs. Government and healthcare setting policies related to ensuring that MHPs have adequate rest may have utility in reducing negative mental health outcomes of workers affected by the COVID-19 pandemic and future public health crises.

## Data Availability

The raw data supporting the conclusions of this article will be made available by the authors, without undue reservation.

## Supplementary material

Supplementary Figure 1 Subgroup analysis of the nonlinear association between fatigue and PTSD risk.

Supplementary Figure 2 Subgroup analysis of the nonlinear association between fatigue and fear of COVID-19.

Supplementary Table 1 Baseline characteristics of included participants compared with non-COVID participants.

Supplementary Table 2 Linear regression analyses of associations between fatigue and PTSD and fear of COVID-19.
