# Supplementary material for: Nonlinear relationships between fatigue, fear of COVID-19, and PTSD among mental health professionals: the findings of a multi-site survey in China
Source: Front Psychiatry. 2026 Apr 10;17:1731508. doi: 10.3389/fpsyt.2026.1731508 (PMC13106409; doi:10.3389/fpsyt.2026.1731508)

**Supplementary materials**

Table S1. Baseline characteristics of included participants compared with non-COVID participants

Table S2. Linear regression analyses of associations between fatigue and PTSD and fear of COVID‑19

Figure S1. Subgroup analysis of the nonlinear association between fatigue and PTSD risk

Figure S2. Subgroup analysis of the nonlinear association between fatigue and fear of COVID-19

Table S1. Baseline characteristics of included participants compared with non-COVID participants

|  | Included (n = 9,858) | Non-COVID (n = 789) | Univariable analysis | |  |
| --- | --- | --- | --- | --- | --- |
|  |
|  |
|  |
| Measure | n (%) | n (%) | χ^2^ | *p* |  |
| Male gender | 1,750 (17.75) | 170 (21.55) | 7.11 | 0.008 |  |
| Married marital status | 7,209 (73.13) | 513 (65.02) | 24.11 | <0.001 |  |
| College or above education level | 9,346 (94.81) | 743 (94.17) | 0.60 | 0.44 |  |
| Perceived Health Status |  |  | 20.50 | <0.001 |  |
| Poor | 653 (6.62) | 45 (5.70) |  |  |  |
| Fair | 7043 (71.44) | 516 (65.40) |  |  |  |
| Good | 2162 (21.93) | 228 (28.90) |  |  |  |
|  |  |  |  |  |  |
|  | Mean (SD) | Mean (SD) | *Z* | *p* |  |
| Age (years) | 34.82 (8.30) | 35.27 (9.47) | -0.07 | 0.941 |  |
| Fatigue | 3.98 (2.44) | 3.45 (2.60) | -6.09 | <0.001 |  |
| Infection duration (weeks) | 0.00 (0.00) | 2.42 (3.12) | - | <0.001 |  |
| Working years (years) | 13.04 (10.23) | 12.65 (9.07) | -0.42 | 0.673 |  |
| Notes: SD: Standard Deviation; 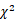: Chi-Square Test; *Z:* Standardized test statistic from the Mann-Whitney U Test. The dash (-) indicates that the test statistic was not applicable due to zero variance in the "COVID-19 Recovered" group. | | | | |  |

Table S2. Linear regression analyses of associations between fatigue and PTSD and fear of COVID‑19

| Dependent Measure | PTSD Total Scores | | |  | FOC Total Scores | | |
| --- | --- | --- | --- | --- | --- | --- | --- |
|  | *p* | *OR* | 95% *CI* |  | *p* | *OR* | 95% *CI* |
| Model1a | <0.001 | 1.92 | 1.86-1.98 | Model1b | <0.001 | 0.72 | 0.68-0.77 |
| Model2a | <0.001 | 1.70 | 1.64-1.77 | Model2b | <0.001 | 0.65 | 0.60-0.70 |
| PTSD:  Model 1b: Adjusted for no covariates;  Model 2b: Adjusted for gender, perceived health status, age, working years and infection duration.  Fear of COVID-19: | | | | | | | |
| Model 1a: Adjusted for no covariates; Model 2a: Adjusted for gender, marital status, education level, perceived health status and infection duration.  Note: CI: confidence interval; FOC: fear of COVID‑19; infection duration: time since COVID‑19 infection; OR: odds ratio; PTSD: post‑traumatic stress disorder. | | | | | | | |

Figure S1. Subgroup analysis of the nonlinear association between fatigue and PTSD risk


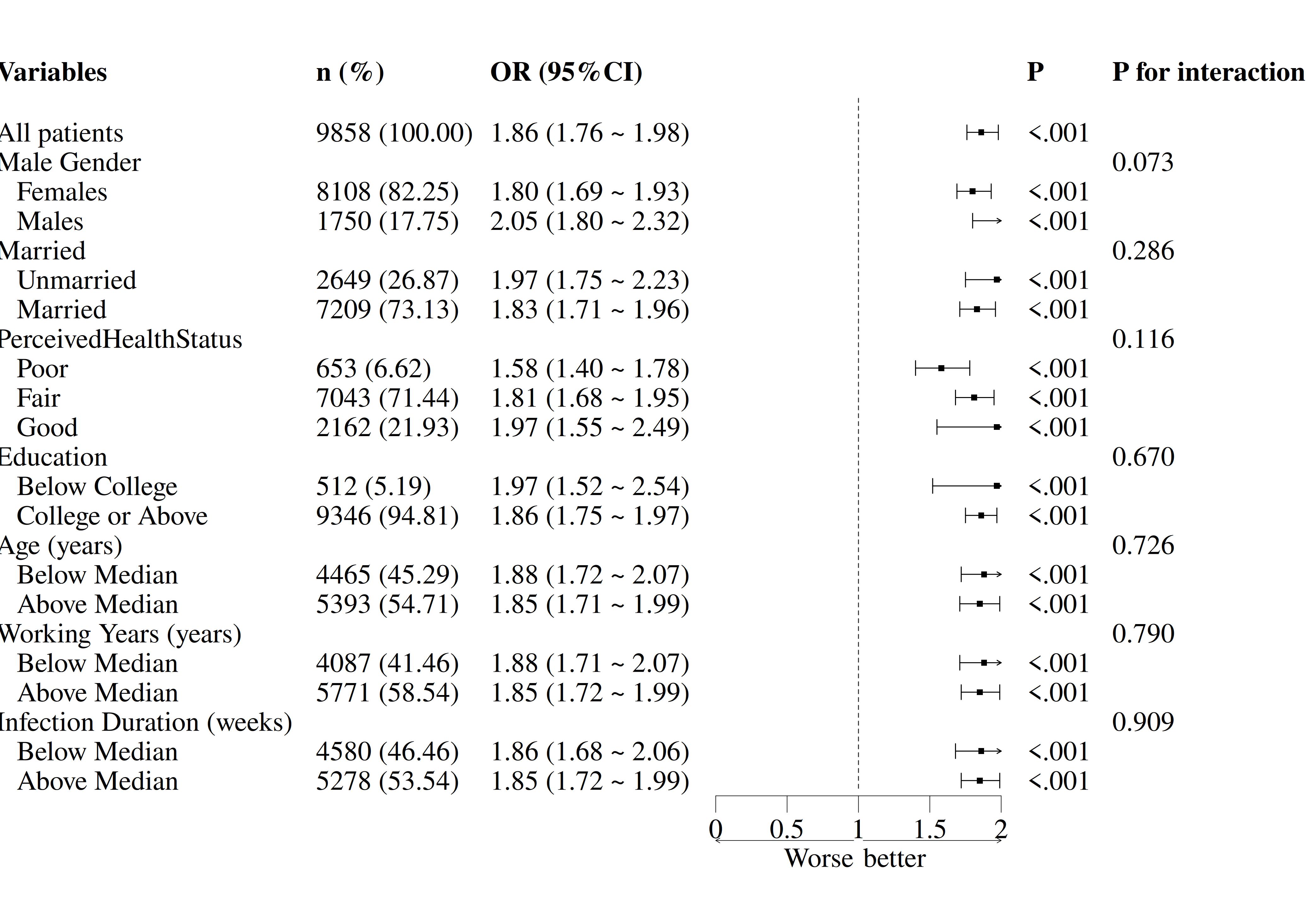


Figure S2. Subgroup analysis of the nonlinear association between fatigue and fear of COVID-19


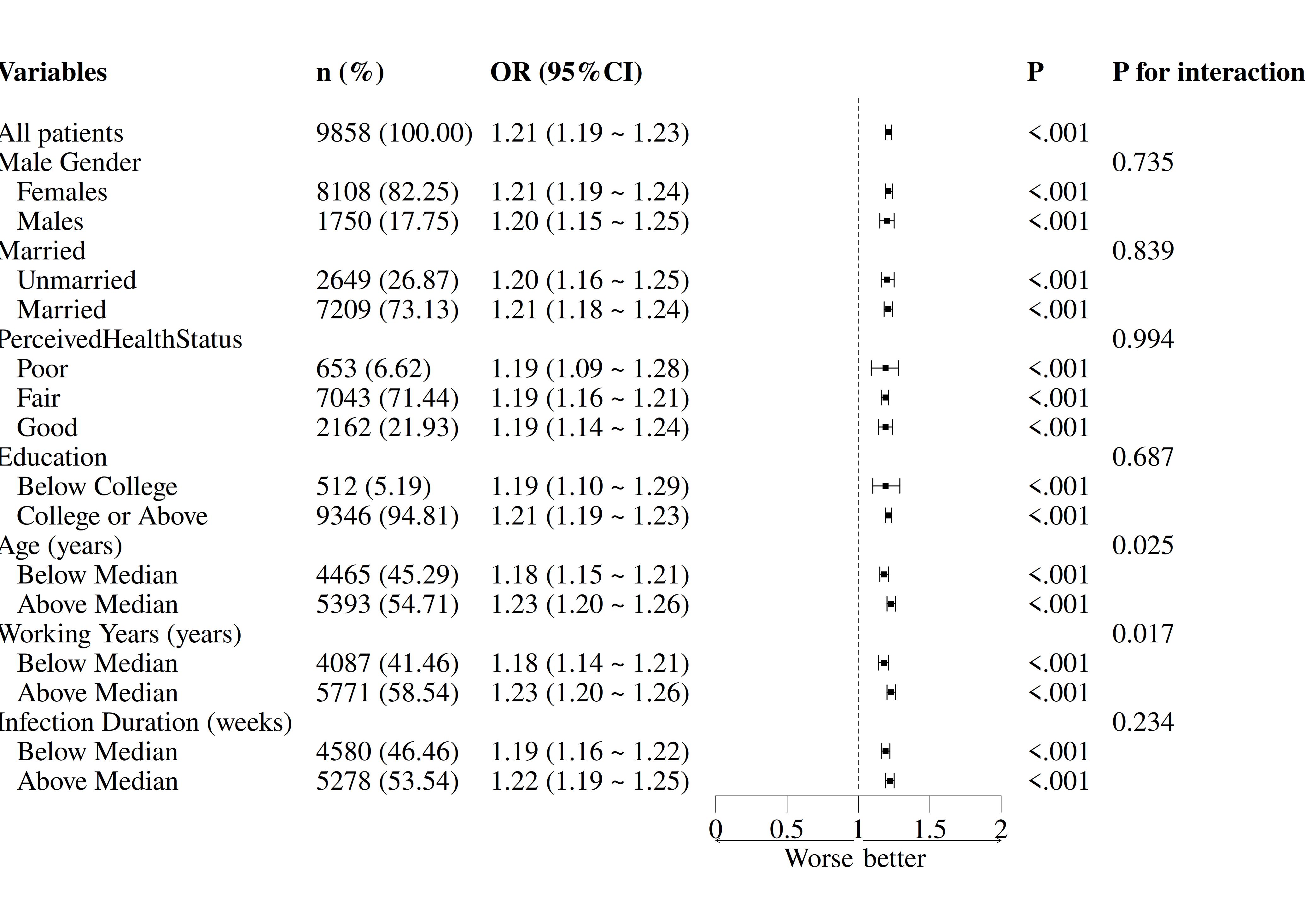

Supplement: Supplementary Figure 1 — Subgroup analysis of the nonlinear association between fatigue and PTSD risk. [file Supplementaryfile1.doc]
